# Quantitative MRI Evaluation of Coracohumeral Ligament and Inferior Glenohumeral Capsule Thickening in Adhesive Capsulitis: Correlation with Range of Motion and Edema Patterns

**DOI:** 10.5152/eurasianjmed.2026.251185

**Published:** 2026-02-02

**Authors:** Seda Soğukpınar Karaağaç, Nevzat Yeşilmen

**Affiliations:** 1 Department of Radiology, Fırat University Faculty of Medicine, Elazığ, Türkiye; 2 Department of Physical Medicine and Rehabilitation, University of Health Sciences, Elazığ Fethi Sekin City Hospital, Elazığ, Türkiye

**Keywords:** Adhesive capsulitis, ligaments, magnetic resonance imaging, physical, radiology, range of motion, shoulder joint

## Abstract

**Background:**

Adhesive capsulitis involves capsular fibrosis and restricted shoulder movements, particularly external rotation and abduction. Magnetic resonance imaging (MRI) enables assessment of capsular and ligamentous thickening. The relationship between capsular thickness, range of motion (ROM), and rotator cuff and biceps tendon pathologies remains unclear. This study aimed to investigate the relationship between the coracohumeral ligament and inferior glenohumeral capsule thickness, shoulder range of motion, and MRI-detected edema patterns in patients with adhesive capsulitis.

**Methods:**

This prospective study included 100 adhesive capsulitis patients who underwent shoulder MRI between July 2024 and July 2025. Coracohumeral ligament (CHL) and inferior glenohumeral capsule (IGHC) thicknesses were measured, and rotator cuff and biceps tendon pathologies graded. Shoulder ROM in external rotation (ER) and abduction was measured using a goniometer. Analyses included correlation tests, multiple linear regression, and Kruskal–Wallis tests.

**Results:**

The mean patient age was 57.7 ± 11.9 years, with 66% women. CHL thickness strongly negatively correlated with ER ROM (*r* = −0.82, *P* < .001), while IGHC thickness strongly negatively correlated with abduction ROM (*r* = −0.79, *P* < .001). Regression analysis showed that CHL thickness independently predicted ER limitation, and IGHC thickness predicted abduction limitation. Rotator cuff and biceps tendon pathologies were common but not independently associated with ROM.

**Conclusion:**

Coracohumeral ligament and IGHC thickening are key determinants of restricted shoulder mobility in adhesive capsulitis. Magnetic resonance imaging assessment of these structures provides reliable diagnostic markers and can guide treatment, while concomitant rotator cuff and biceps pathologies have a limited impact on ROM restriction.

**Main Points**

- Adhesive capsulitis is primarily characterized by thickening of the coracohumeral ligament (CHL) and inferior glenohumeral capsule (IGHC).
- Coracohumeral ligament thickening strongly correlates with external rotation loss, while IGHC thickening correlates with abduction loss.
- Magnetic resonance imaging (MRI)-based quantitative measurements of CHL and IGHC thickness are reliable markers of disease severity.
- Rotator cuff and biceps tendon pathologies are common but do not independently contribute to range of motion restrictions.
- Magnetic resonance imaging findings should be integrated with clinical evaluation to improve treatment planning for adhesive capsulitis.

## Introduction

Adhesive capsulitis is a shoulder pathology characterized by progressive fibrosis and inflammation of the glenohumeral joint capsule, commonly referred to as a frozen shoulder. Although its exact etiology remains unclear, it predominantly affects middle-aged individuals, particularly women and patients with diabetes. The clinical symptoms are typically pain and severe shoulder movement restriction, particularly during external rotation and abduction.^1^^,^^2^

Magnetic resonance imaging (MRI) is an important diagnostic method for detecting early inflammatory changes and capsule thickening associated with adhesive capsulitis. Previous studies have emphasized that coracohumeral ligament (CHL) thickness, particularly on fat-suppressed T2-weighted images, is an important imaging marker of adhesive capsulitis. Similarly, inferior glenohumeral capsule (IGHC) thickness is considered a reliable indicator of capsular contracture severity.^3^^-^^5^ Despite extensive research on capsular and ligamentous changes, the relationship between adhesive capsulitis and accompanying rotator cuff tendon pathologies has not been sufficiently investigated. Understanding this relationship is crucial because rotator cuff pathology can complicate clinical findings and potentially influence treatment strategies.^6^^,^^7^ This study aimed to address gaps in the current clinical knowledge by comprehensively investigating the presence of accompanying edema at the axillary recess and rotator interval levels, CHL and IGHC thicknesses, shoulder range of motion (ROM) limitations, and the relationship between rotator cuff and biceps tendon pathologies in a large group of patients with adhesive capsulitis.

## Material and Methods

### Patients

This study was approved by the Fırat University Non-Interventional Research Ethics Committee (Decision No. 34460, Date: May 14, 2025). This study was conducted between July 2024 and July 2025 at Fırat University Hospital in patients diagnosed with adhesive capsulitis based on shoulder MRI and clinical evaluation. Patients aged ≥ 18 years with shoulder complaints lasting for at least 6 months were included in the study. Of the 173 patients who met the criteria, 73 were excluded from the study due to previous shoulder surgery, shoulder trauma involving fracture or dislocation, calcific tendinitis, septic arthritis, osteoarthritis, labral pathology, neoplastic conditions, neurological deficits, clinical-radiological inconsistencies, or insufficient medical records and patients who could not be examined. The demographic characteristics, clinical findings, and known diseases of the 100 patients diagnosed with adhesive capsulitis included in the study were evaluated and recorded.

### MRI Protocol

Shoulder MRI examinations were performed using a 1.5T or 3T MRI (Intera Achieva, Philips Healthcare) device with a special shoulder coil. During the examination, the patients were imaged in the supine position with their arms turned outward. The following imaging parameters were used: oblique coronal fat-suppressed T2-weighted imaging (TR/TE, 2700-3700/64-73; echo train length, 10-16; slice thickness, 3 mm; matrix, 480 × 480; FOV, 14-15 cm), oblique coronal T1-weighted imaging (TR/TE, 520-780/11-19; echo train length, 2-4; slice thickness, 2.5 mm; matrix, 512 × 512; FOV, 14 cm), oblique sagittal fat-suppressed T2-weighted imaging (TR/TE, 3200-4300/72-90; echo train length, 12-18; slice thickness, 2.5-3 mm; matrix, 512 × 512; FOV, 14 cm), axial fat-suppressed T2-weighted imaging (TR/TE, 2800-3600/73-90; echo train length, 10-18; slice thickness, 3-3.5 mm; matrix, 512 × 512; FOV, 14 cm).

Both 1.5T and 3T MRI datasets were included in the study. Although 3T imaging provides a higher SNR, the ligament–capsule boundaries were clearly discernible at both field strengths. No statistically significant difference was found between the measurements obtained on the 1.5T and 3T systems (*P* > .05). Therefore, the measurements were comparable across the field strengths.

### Imaging Analysis

All radiological examinations were assessed by a board-certified radiologist with 13 years of experience. Both qualitative and quantitative variables pertinent to the diagnosis of adhesive capsulitis were identified as delineated in the literature.^3^^-^^5^^,^^8^^,^^9^

#### Qualitative Analyses

**Figure 1.**
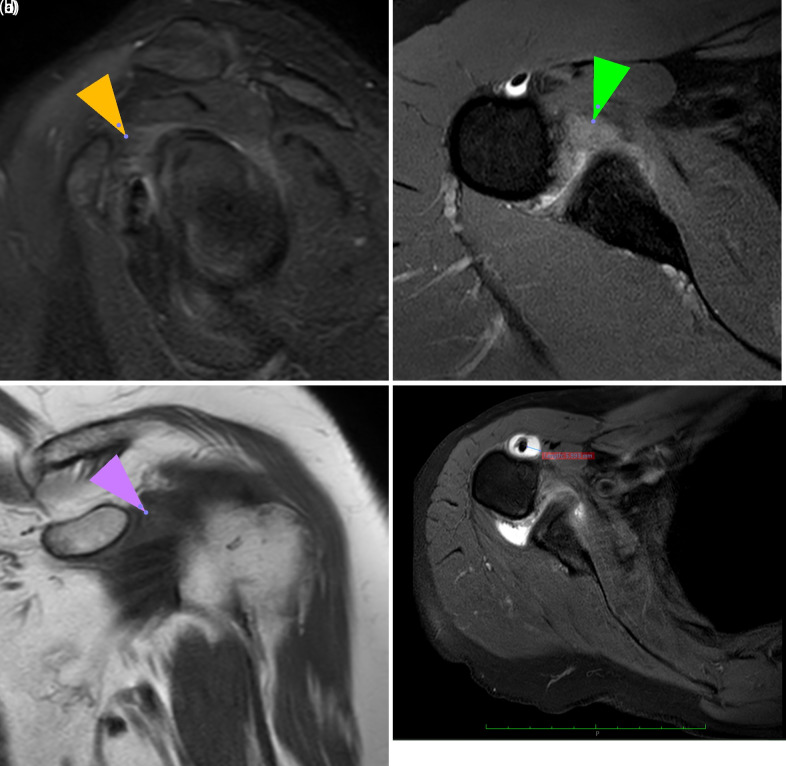
a. Edema observed in the sagittal T2-weighted fat-suppressed MR image at the rotator interval (yellow arrow). b. Edema observed in the axial T2-weighted fat-suppressed MR image at the axillary recess level of the joint capsule (green arrow). c. Fat tissue obliteration observed in the subcoracoid area on coronal T1-weighted MR image (purple arrow). d. Fluid surrounding the long head of the biceps tendon in the bicipital groove at the humeral neck (>2 mm) on axial T2-weighted fat-suppressed MR image.

The presence or absence of the following findings was evaluated: edema at the rotator interval level, which is the region between the subscapularis and supraspinatus tendons through which the long head of the biceps tendon passes and where the coracohumeral and superior glenohumeral ligaments are situated, as well as at the axillary recess joint capsule level; obliteration of the subcoracoid fat pad; staged rotator cuff tendon pathologies; and tendinitis and effusion of the long head of the biceps tendon within the groove. Edema at the axillary recess joint capsule and rotator interval levels was correlated and identified on oblique sagittal, coronal, and axial fat-suppressed T2-weighted MR images (Figure 1a and b). The obliteration of the subcoracoid fat pad is characterized by a reduction in signal intensity relative to that of the subcutaneous fat at the same level on the oblique coronal T1-weighted images. Both partial and complete obliteration are indicative of adhesive capsulitis^10^^-^^13^ (Figure 1c). Effusion at the bicipital groove of the long head of the biceps tendon is deemed clinically significant when the fluid depth surrounding the tendon exceeds 2 mm on axial fat-suppressed T2-weighted MR images at the humeral neck level (Figure 1d). Rotator cuff tendon pathologies were assessed by correlating the findings across the axial, coronal, and sagittal fat-suppressed T2-weighted sequences. Tendon abnormalities involving the supraspinatus, infraspinatus, and subscapularis were graded on a 0-4 scale. Stage 0 represents normal tendon morphology. Stage 1 indicates mild tendinosis with minimal T2 hyperintensity. Stage 2 reflects moderate tendinosis, characterized by increased signal intensity and early fiber disruption. Stage 3 corresponded to severe tendinosis with marked architectural distortions. Stage 4 represents partial- or full-thickness tendon tears.^10^^-^^12^

#### Quantitative Analyses

**Figure 2.**
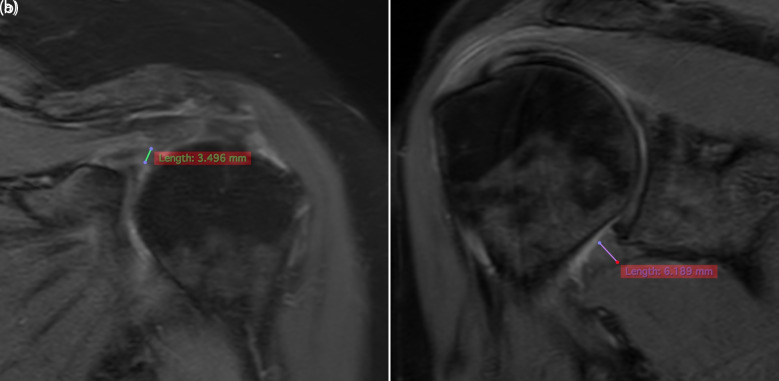
a. Measurement of the thickest slice of the coracohumeral ligament (CHL) in the coronal T2-weighted fat-suppressed MR image at the rotator interval level (3.49 mm). b. Measurement of the inferior glenohumeral capsule (IGHC) thickness at the axillary recess level in the coronal T2-weighted fat-suppressed MR image from the thickest edematous slice (6.1 mm).

Measurements were performed using a linear and perpendicular method. The CHL thickness was measured on the coronal fat-suppressed T2 sequence at the rotator interval, from the base of the coracoid process to the thickest visible portion of the ligament (Figure 2a). The IGHC thickness was measured at the axillary recess on the coronal fat-suppressed T2 sequence, perpendicular to the capsular surface at its thickest point (Figure 2b). All measurements were obtained from the slices in which the anatomical borders were most clearly visualized.

### Clinical Evaluation

All patients underwent a physical examination performed by a Physical Medicine and Rehabilitation specialist with 14 years of experience. Informed consent was obtained from each patient prior to clinical examination. Passive shoulder ROM was assessed using a universal goniometer. The diagnosis of adhesive capsulitis was based on the clinical criteria defined by Codman. The main Codman criteria used in the diagnosis were the presence of shoulder pain lasting longer than 6 months and particularly disturbing night sleep, at least 20° limitation in passive external rotation compared to the contralateral shoulder (if both shoulders are affected, 45° is accepted as the reference angle), and no pathological findings on conventional radiographs.^14^^,^^15^

During the clinical examination, the limitation in shoulder abduction was assessed using external rotation, which is the first movement affected by adhesive capsulitis. The intraclass correlation coefficient (ICC) for ROM measurements was previously determined to be 0.93. External rotation was measured as the maximum angle achieved by externally rotating the shoulder while the elbow was flexed at 90° and the arm was in the neutral position. The degree of abduction was determined by having the patients raise their arms laterally to the full abduction (180°) position above their heads.

### Statistical Analysis

Data analysis was conducted using SPSS version 25.0 for Windows. The Kruskal–Wallis test was used to examine the correlations between the quantitatively assessed MRI findings. Statistical significance was set at *P* < .05. For qualitative MRI findings, the Pearson/Spearman correlation coefficient (*r*) was utilized. The “*r*” value ranges from −1 to +1, with values approaching +1 indicating a stronger positive correlation and values approaching −1 indicating a stronger negative correlation.

## Results

The mean age of the patients was 57.7 ± 11.9 (27-92); 66% were female, and 32% were male. The right arm was the most commonly affected (58%). The demographic characteristics are summarized in Table 1.

**Figure 3.**
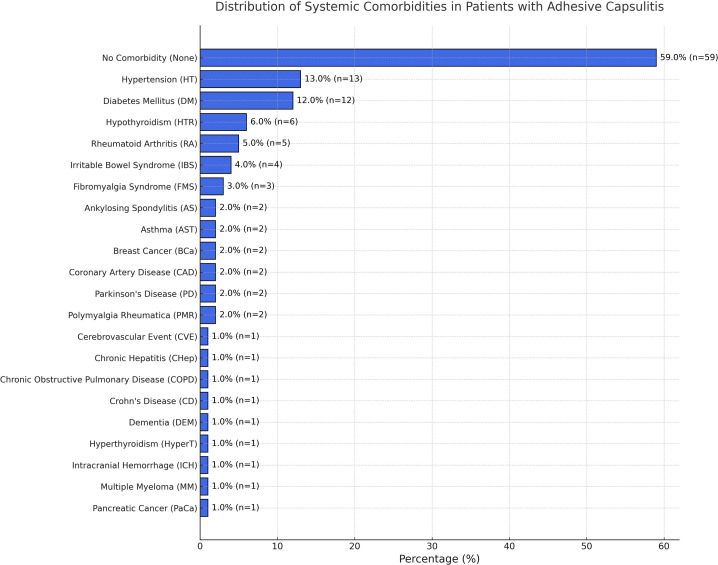
Distribution of systemic diseases in patients with adhesive capsulitis. Hypertension and diabetes mellitus were the most common comorbidities.

The most common comorbidities were hypertension, diabetes mellitus, and hypothyroidism. These were analyzed both per patient and in terms of total case numbers (including accompanying diseases) (Figure 3). It was observed that 34% of the patients had at least 1 chronic disease. Among these, diabetes mellitus (12%) and hypertension (13%) were the most prevalent.

In the statistical analysis performed, the mean CHL thickness was 4.20 ± 0.99 mm (2.1-5.5 mm), and the IGHC thickness was 4.15 ± 0.92 mm (2.6-6 mm). The mean external rotation ROM was 53.8 ± 12.4° (30-80°), and the abduction ROM was 121.7 ± 29.2° (40-175°). The findings are summarized in Table 2.

**Figure 4.**
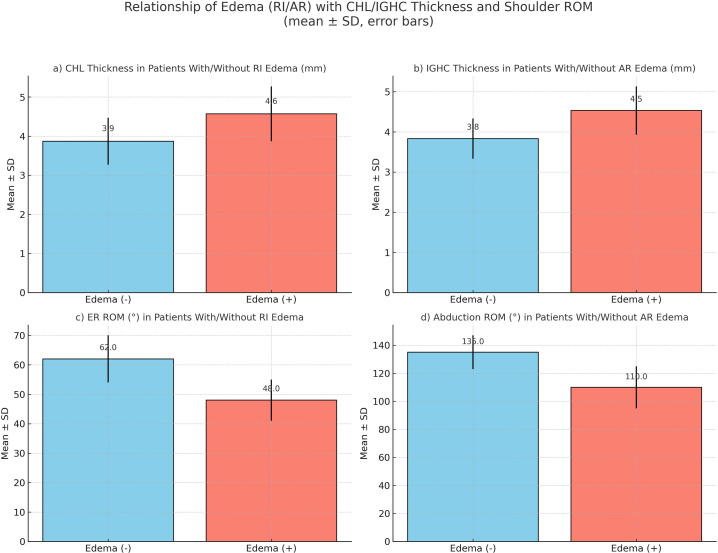
Correlation between CHL/IGHC thickness and range of motion (ROM). a. Strong negative correlation between CHL thickness and external rotation. b. Moderate negative correlation between CHL thickness and abduction. c. Moderate negative correlation between IGHC thickness and external rotation. d. Strong negative correlation between IGHC thickness and abduction.

In patients with adhesive capsulitis, a significant decrease in external rotation (ER) ROM was observed as the CHL thickness increased (*r* = −0.821, *P* < .001). A moderate negative correlation was found between CHL thickness and abduction ROM (*r* = −0.376, *P* < .001). Patients with increased IGHC thickness had decreased abduction ROM, and a strong negative correlation was found between them (*r* = −0.794, *P* < .001). A mild-to-moderate level of movement restriction was observed in the ER group as IGHC thickness increased (*r* = −0.316, *P* = .0014). Multivariate regression analysis showed that CHL thickness independently predicted decreased ER ROM and IGHC thickness independently predicted decreased abduction ROM (Figure 4).

**Figure 5.**
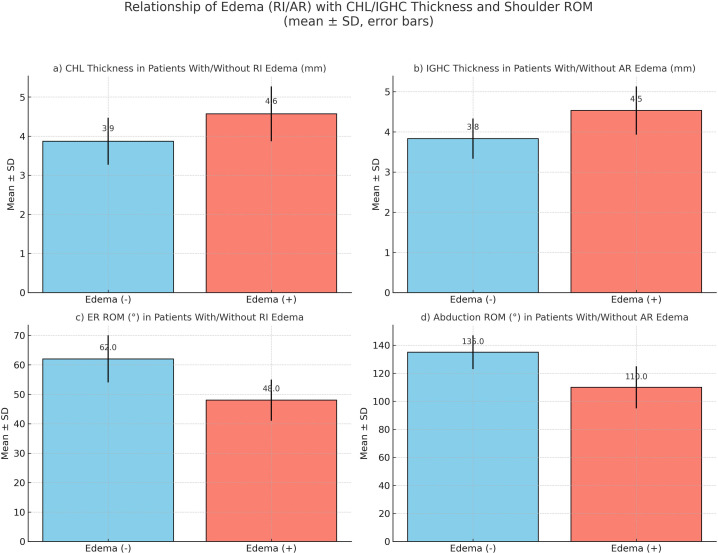
Relationships between edema at the rotator interval (RI) and axillary recess (AR) levels, CHL/IGHC thickness, and ROM. a. CHL thickness tends to be higher in patients with RI edema. b. IGHC thickness tends to be higher in patients with AR edema. c. RI edema is associated with greater limitation in external rotation. d. AR edema is associated with greater limitation in abduction.

The presence of edema at the axillary recess (AR) and rotator interval (RI) levels and related findings among the anatomical structures evaluated when diagnosing adhesive capsulitis were examined using statistical analysis. In patients with edema detected at the AR level, IGHC thickness significantly increased and abduction range of motion markedly decreased (*P* < .001). Similarly, in patients with edema at the RI level, a significant increase in CHL thickness and a marked decrease in the range of external rotation were detected (*P* < .001). These findings suggest that the presence of edema may be associated with capsular thickening and functional limitations (Figure 5). No statistically significant relationship was found between the presence of edema in the aforementioned regions and any other parameters.

Approximately 54% of patients with adhesive capsulitis had pathologies in the long head of the biceps tendon. The most common pathological finding was effusion in the bicipital groove (32%)**, **followed by tendinitis (19%) and, rarely, partial tears (3%). Although no statistical relationship was found with other parameters, effusion was considered a finding associated with adhesive capsulitis.

**Figure 6.**
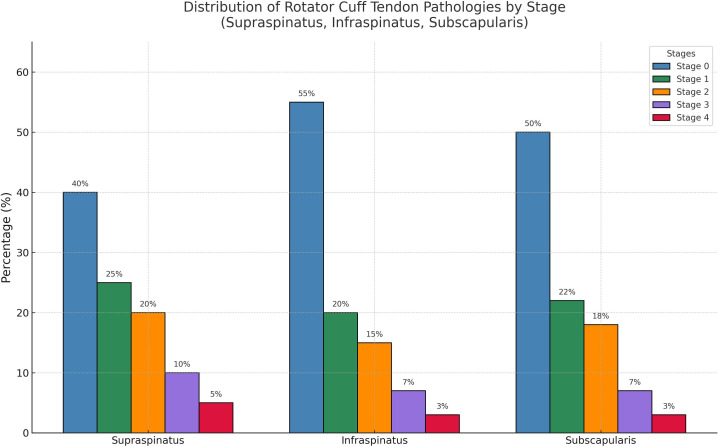
Distribution of rotator cuff tendon pathologies by stage in patients with adhesive capsulitis. Normal tendon morphology (stage 0) was the most common finding, followed by early stage tendinopathy (stages 1-2), whereas advanced degeneration (stages 3-4) was rare.

The frequency and stages of rotator cuff tendon pathologies were evaluated in patients with adhesive capsulitis. Stage 0 (normal) was the most common group, with no structural deterioration observed in all tendons in a significant proportion of the patients. However, degenerative findings, such as stage 1 and 2 tendinopathy, were more commonly detected, particularly in the supraspinatus and subscapularis tendons. Stages 3 and 4 (advanced degeneration) were rarely observed in any tendon (Figure 6). These findings indicate that most patients with adhesive capsulitis have accompanying pathologies in the rotator cuff tendons that have not progressed to an advanced stage. These results support the notion that low-to-moderate pathologies in the rotator cuff tendons are not directly related to the ROM limitations seen in adhesive capsulitis.

## Discussion

This study investigated morphological and functional changes in adhesive capsulitis through detailed MRI analysis and clinical correlation. Specifically, the role of CHL and IGHC thickening was examined, and their relationship with joint ROM, AR, and RI edema was evaluated. The results demonstrated that CHL and IGHC thickening were strongly correlated with external rotation and abduction restrictions, respectively. This central finding establishes a basis for further comparison with previous studies.

In the cohort, adhesive capsulitis was more common in women (66%), and the right shoulder was slightly more frequently involved (58%). This finding is consistent with previous studies that reported a higher prevalence in women and an association with endocrine and hormonal factors.^2^^,^^15^ The predominance of right-sided involvement may be explained by the dominant extremity being more susceptible to repetitive microtrauma or increased mechanical stress, as suggested in earlier literature.^13^

The presence of systemic comorbidities, particularly diabetes mellitus (12%) and hypertension (13%), is noteworthy. Diabetes mellitus has long been recognized as a major risk factor for adhesive capsulitis, with a prevalence rate of up to 20% among patients with diabetes mellitus.^2^^,^^16^ Chronic hyperglycemia and advanced glycation end-products have been implicated in the promotion of capsular fibrosis through cytokine-mediated mechanisms. Similarly, hypothyroidism and other metabolic disorders may contribute to collagen deposition and tissue contracture, supporting the notion that systemic diseases increase the risk of developing adhesive capsulitis.^16^ The coexistence of multiple comorbidities in some patients in the study further supports this relationship.

The results demonstrated a strong negative correlation between CHL thickness and external rotation and between IGHC thickness and abduction. Multivariate regression confirmed that each structure independently predicted specific motion limitations, underscoring their complementary roles in functional impairment.

However, previous studies have presented conflicting results regarding these relationships. Kerimoğlu et al in a study with a small sample size (n = 17), did not find a significant correlation between CHL thickness and ROM.^17^ In contrast, Inada et al reported a significant negative correlation between IGHC thickness and all ranges of shoulder motion, including abduction and external rotation, using ultrasonography.^6^ Similarly, Guity et al confirmed that capsular contracture and thickening were closely associated with ROM limitations, particularly in the axillary recess.^7^ This study, with a larger sample size, confirms and extends these findings by showing that CHL thickening is the primary determinant of external rotation loss, whereas IGHC thickening is the main factor restricting abduction. This anatomical-functional correlation may help explain the typical clinical presentation of adhesive capsulitis, where external rotation is the earliest and most severely affected movement, followed by abduction.

Another important finding was the association between edema in the AR and RI with structural thickening and motion loss. Patients with AR edema demonstrated significantly thicker IGHC and reduced abduction ROM, whereas patients with RI edema had increased CHL thickness and greater external rotation restriction. These findings support the concept that edema represents an early inflammatory phase of adhesive capsulitis that may evolve into fibrosis and contracture.

Chi et al reported that axillary recess and rotator interval changes had relatively low sensitivity but high specificity for adhesive capsulitis diagnosis.^18^ Lee et al also emphasized that AR edema is a prominent feature of the early inflammatory stage.^10^ The results are consistent with these reports and highlight that MRI-based identification of edema can provide additional insight into the disease phase and inflammatory activity. This is particularly relevant for treatment planning, as patients in the inflammatory stage may respond better to anti-inflammatory therapy or intra-articular injections, whereas those in the fibrotic stage may require more aggressive physical therapy or even surgical intervention.

In this study, approximately 54% of patients had concomitant pathologies of the long head of the biceps tendon, most frequently effusion (32%) and tendinitis (19%). Partial tears were rare (3 cases). Although these findings were common, they did not show a significant relationship with ROM restrictions. This supports the idea that biceps involvement is an accompanying phenomenon rather than a direct determinant of functional loss.

Similarly, rotator cuff tendon pathologies, particularly low-grade degeneration of the supraspinatus and subscapularis tendons, were observed in a notable proportion of patients. However, advanced degeneration or complete tears were rare. These results align with those of Sharma et al and others, who reported that mild tendinopathy is often observed in adhesive capsulitis but does not directly correlate with motion loss.^11^^,^^12^ Therefore, these findings reinforce the conclusion that, while rotator cuff and biceps pathologies are frequent, they are not the primary cause of restricted mobility in patients with adhesive capsulitis.

This study has several limitations. The sample size was relatively small, and due to the cross-sectional design, disease progression over time could not be assessed. Only passive abduction and external rotation were evaluated; internal rotation and flexion, which may also correlate with the MRI findings, were not analyzed. Furthermore, some MRI features described in the literature, such as subtle capsular distension, synovial hypertrophy, and rotator interval scarring, were not included in the evaluation. All measurements were performed by a single radiologist, and the lack of inter-observer reliability testing represents another limitation. Finally, the study was conducted at a single center, which may limit the generalizability of the results. Prospective multicenter studies with larger cohorts and clinical staging are needed to confirm and expand these observations.

Taken together, these results highlight that MRI-based quantitative assessment of CHL and IGHC thickness, combined with edema evaluation, offers objective and reproducible markers for the diagnosis and treatment planning of adhesive capsulitis.

Adhesive capsulitis is primarily characterized by capsular and ligamentous thickening. Quantitative MRI measurements of the CHL and IGHC, along with edema patterns in the rotator interval and axillary recess, provide reliable imaging markers that support diagnostic evaluation and inform clinical decision making. Although rotator cuff and biceps tendon abnormalities were frequently observed, they did not show an independent association with range-of-motion limitation and therefore appeared to be accompanying rather than determinative findings. These results emphasize that capsular pathology plays a central role in the functional restriction of adhesive capsulitis.

## Figures and Tables

**Table 1.** Demographic Characteristics of Patients With Adhesive Capsulitis

**Variable**	**Value**
Mean age, years	57.7 ± 11.9
Female, %	66.0
Male, %	34.0
Right arm, %	58.0
Left arm, %	42.0

**Table 2.** Capsule–Ligament Thicknesses and Shoulder Range of Motion (ROM) in Patients with Adhesive Capsulitis

**Parameter**	**Mean ± SD**
CHL thickness, mm	4.20 ± 0.99
IGHC thickness, mm	4.15 ± 0.92
External rotation ROM, °	53.8 ± 12.4
Abduction ROM, °	121.7 ± 29.2

CHL, coracohumeral ligament; IGHC, inferior glenohumeral capsule; ROM, range of motion.

## Data Availability

The data that support the findings of this study are available on request from the corresponding author.
